# ﻿*Huperziacrassifolia* (Lycopodiaceae), a new species from China based on morphological characters and molecular evidence

**DOI:** 10.3897/phytokeys.246.131046

**Published:** 2024-08-28

**Authors:** Zhi-You Guo, Hong-Mei Liu, Kai-Kai Wang, Tao Fujiwara, Zheng-Yu Liu, Xian-Chun Zhang, Harald Schneider

**Affiliations:** 1 College of Biological Sciences and Agriculture, Qiannan Normal College for Nationalities, Duyun 558000, Guizhou, China Qiannan Normal College for Nationalities Duyun China; 2 Center for Integrative Conservation & Yunnan Key Laboratory for Conservation of Tropical Rainforests and Asian Elephants, Xishuangbanna Tropical Botanical Garden, Chinese Academy of Sciences, Mengla, Menglun 666303, Yunnan, China Xishuangbanna Tropical Botanical Garden, Chinese Academy of Sciences Mengla China; 3 University of Chinese Academy of Sciences, Beijing 100049, China University of Chinese Academy of Sciences Beijing China; 4 Current address: The Mt. Fuji Institute for Nature and Biology, Showa University, 4562 Kamiyoshida, Fujiyoshida, Yamanashi 403-0005, Japan Showa University Fujiyoshida Japan; 5 Chongqing Institute of Medical Plant Cultivation, Nanchuan 408435,Chongqing, China Chongqing Institute of Medical Plant Cultivation Chongqing China; 6 State Key Laboratory of Systematics and Evolutionary Botany, Institute of Botany, Chinese Academy of Science, Beijing 100093, China Institute of Botany, Chinese Academy of Science Beijing China

**Keywords:** Firmoss, integrative taxonomy, micromorphology, phylogenomics

## Abstract

A new species of the firmoss from China, *Huperziacrassifolia* sp. nov., is described and illustrated based on morphological characters and molecular evidence. The new species resembles species associated with the *H.javanica* complex, in particular *H.javanica* based on leaf shape and serrations, but can be easily distinguished by elliptic lanceolate and thick coriaceous leaves, well differentiated seasonal constriction zones, and reflexed leaf margins when get dried. Phylogenomic reconstruction using whole chloroplast genome sequences recovered *H.crassifolia* as sister to *H.sutchueniana* and only distantly related to morphological similar species *H.javanica*, *H.nanlingensis*, and *H.serrata.* The genome size 2C = 17.2 pg indicated the new species to be a tetraploid, whereas diploid *H.javanica* had a genome size of 8.7 pg. Morphological characters, distribution, and conservation status of the new species are also presented.

## ﻿Introduction

*Huperzia* Bernh. is a genus of about 25 species (PPG I 2016) that occurs mainly in temperate and boreal climatic zones of the world (Zhang and Iwatsuki 2013; Field et al. 2016). Its taxonomic boundary with *Phlegmariurus* Holub has long been controversial (Holub 1964, 1985; Ching 1978, 1981; Wikström and Kenrick 1997; Zhang and Kung 2000; Zhang 2004; Christenhusz et al. 2011) and now an agreement was made about the segregation of these two genera (Zhang and Iwatsuki 2013; Field et al. 2016; PPG I 2016; Chen et al. 2021). Taxonomic uncertainty is not only restricted to the generic classification but affects the estimation of the species diversity that is expected still to be underestimated due to the difficulty in the taxonomy of this genus despite significant progress having been achieved (Zhang and Kung 2000; Zhang and Iwatsuki 2013; Shrestha et al. 2014; Lim and Sun 2015; Lim et al. 2015; Shrestha and Zhang 2015a, b; Testo et al. 2016).

Taxonomic treatments of the Southeast Asian species of *Huperzia* have mainly relied on morphology, including the shape of the trophophylls, serrations, leaf petiole, and ratio of width to length (Ching 1981; Yang 1989, 1990; Zhang and Kung 2000; Zhang and Iwatsuki 2013; Shrestha and Zhang 2015b; Shalimov et al. 2017), and still rarely combined with molecular evidence (Shrestha et al. 2014; Shrestha and Zhang 2015a). Recent taxonomical efforts have clarified the status of several local and confusing species, including the segregation of *H.asiatica* from *H.lucidula* (Lim et al. 2015; Shrestha and Zhang 2015a) and *H.continentalis* from *H.haleakalae* (Testo et al. 2016). Two new species *H.jejuensis* (Lim and Sun 2015) and *H.nanlingensis* (Shrestha et al. 2014) were discovered from Korea and China, respectively. However, species delimitation has been challenging because of the very subtle differences in the gross morphology among species. Besides the subtle differences among morphologically similar taxa in *Huperzia*, hybrids may also hinder the recognition of distinct species (Testo et al. 2016; Vejvodová et al. 2024).

As has previously been recognized, *Huperziaserrata* is the most widely distributed and common *Huperzia* species in China (Ching 1981; Zhang and Kung 2000; Zhang 2004; Zhang and Iwatsuki 2013; Shrestha and Zhang 2015b). Species delimitation of *H.serrata* and its relatives was substantially improved by the efforts of several researchers who separated *H.crispata* and *H.javanica* from the broad species concept *H.serrata* (Ching 1981; Yang 1982; Lim et al. 2015; Shrestha and Zhang 2015b) as well as the recognition of the new species *H.nanlingensis* (Shrestha et al. 2014). Geographically, *H.javanica* occurs in areas along the Yangtze River and throughout southern China, while the distribution range of *H.serrata* is restricted to northeastern China (Shrestha and Zhang 2015b; Chen 2021). However, species delimitation of the *H.javanica* complex as well as other lineages of *Huperzia* remains unclear and awaits more efforts (Chen 2021; Vejvodová et al. 2024).

During medicinal plant inventories in Guizhou, we discovered two morphological distinct forms of *Huperziajavanica* which usually occur together. The gross morphology segregates one of these forms as typical *H.javanica*; the other form is considered as a distinct taxon. This proposal was studied by consulting *Huperzia* checklists of China, local floras, and careful comparison of morphological characters with previously described species by checking specimens and species protologue for all the known species of *Huperzia*. After all these taxonomical revisions, we accept this taxon to represent an undescribed new species. Genome size measurement and phylogenomic reconstruction using whole chloroplast genome sequence were employed to provide additional evidence to support this hypothesis and detect the phylogenetic relationship of this new taxon. Here, we describe this new species based on molecular, macro-, and micro-morphological evidence.

## ﻿Materials and methods

### ﻿Morphological observation

The specimens of the new taxon were collected from Duyun, Guizhou Province, Southwestern China, and field investigations were conducted in 2016. Careful comparison of morphological characters with previously described species was carried out at HITBC, KUN, PYU, and IMC (herbaria codes according to Thiers 2024) as well as on the digitalized specimens from online Chinese Virtual Herbarium (CVH). In order to identify the distinction of the new taxon in the genus *Huperzia*, we checked not only species with similar gross morphology, including *H.javanica* complex but also several congeneric taxa which co-occur in this area (Ching 1981; Zhang and Iwatsuki 2013; Shrestha and Zhang 2015b).

All studied accessions were checked and identified using the treatments provided in the Flora Reipublicae Popularis Sinicae (Zhang 2004) and the Flora of China (Zhang and Iwatsuki 2013) besides specific taxonomic treatments (Ching 1981; Yang 1989; Shrestha and Zhang 2015b). Gross morphology was compared among individuals of the new taxon and accessions of putative relatives such as *H.javanica* and *H.nanlingensis* by analyzing either freshly collected material or images of specimens including type specimens of both species. Several diagnostic characters were carefully checked and compared among all accessions. Several individuals of the new taxon from different localities and its similar species *H.javanica* were sampled to obtain a comprehensive coverage. The voucher specimens of the new species were deposited in the Herbarium of Qiannan Normal College for Nationalities (QNUN).

### ﻿Spore morphology and ornamentation

Scanning electron microscopy (SEM) was used for spore ornamentation for the new taxon and its close relatives *Huperziajavanica*. In preparation, spores were taken from mature sporangia, fixed on carbon tape, and sputtered with gold. The morphology of spores was observed with a scanning electron microscope (Zeiss Evo LS10). The description of spore ornamentation followed Wang and Yu (2003) and Punt et al. (2007).

### ﻿Assessing ploidy via genome size measurement

Living plants from the type locality were cultivated at Xishuangbanna Tropical Botanical Garden, CAS. Fresh leaves were removed from the cultivated plants and used to generate DNA C-value measurement using propidium iodide DNA staining and nuclei counting with a flow cytometer as described previously (Clark et al. 2016; Fujiwara et al. 2023). Leaf fragments were co-chopped together with an internal standard, *Viciafaba* ‘Inovec’ (2C = 26.90 pg) (Doležel et al. 1992) in General Purpose Buffer (GPB) as isolation buffer (Loureiro et al. 2006). The fluorescence intensities were analyzed on BD FACSVerseTM (BD Biosciences, San Jose, CA, USA). These reported measurements were obtained by estimating the average of measurements carried out for three leaf fragments obtained from three individuals (Table 1). These individuals were all collected from the type location. The interpretation of the obtained genome size values was carried out in the context of previously published estimates with special attention to recently published estimates of different cytotypes belonging to *Huperziaselagos.l.* (Vejvodová et al. 2024).

**Table 1.** Genome size measurement of *H.crassifolia* and its relatives. For newly generated genome size measurement, we provide besides the holoploid genome size (2C), the following information: CV (average of the repeatedly measured cytometric values), voucher, extraction buffer, internal calibration standard. For measurements obtained from the literature, we provide the reference instead. The ploidy interpretation assumes a conserved positive correlation between chromosome number and genome size (Clark et al. 2016; Fujiwara et al. 2023; Vejvodová et al. 2024). 2C-values between 8 to 12 pg are considered to be diploid, whereas 2C-values between 16 and 18 pg are considered to be tetraploid.

Taxon	2C (pg)	CV	Voucher or Publication	Buffer	Calibration	Ploidy interpretation
*H.crassifolia*	17.6	0.07	China, Guizhou/LHM2571A	GPB	*Viciafaba* ‘Inovec’	4×
*H.crassifolia*	16.9	0.06	China, Guizhou/LHM2571B	GPB	*Viciafaba* ‘Inovec’	4×
*H.crassifolia*	17.1	0.12	China, Guizhou/ LHM2571C	GPB	*Viciafaba* ‘Inovec’	4×
*H.javanica*	8.6	0.13	China, Guizhou/LHM2569C	GPB	*Viciafaba* ‘Inovec’	2×
*H.javanica*	8.6	0.05	China, Guizhou/LHM2569B	GPB	*Viciafaba* ‘Inovec’	2×
*H.javanica*	8.5	0.04	China, Guizhou/LHM2569A	GPB	*Viciafaba* ‘Inovec’	2×
*H.javanica*	9.2	0.03	China, Guizhou/LHM2572	GPB	*Viciafaba* ‘Inovec’	2×
*H.nanlingensis*	8.7	0.06	China, Guizhou/LHM2568	GPB	*Viciafaba* ‘Inovec’	2×
*H.lucidula*	11.4	NA	Wang et al. (2005)	NA	NA	2×
*H.lucidula*	9.6	NA	Bai et al. (2012)	NA	NA	2×
*H.lucidula*	11.3	NA	Bainard et al. (2011)	NA	NA	2×
*H.selago*	17.3	NA	Šmarda et al. (2019)	NA	NA	4×
*H.selago*	10.0	NA	Vejvodová et al. (2024)	OTTO	*Pisumsativum* ‘Ctirad’	2×
*H.selago*	13.5	NA	Vejvodová et al. (2024)	OTTO	*Pisumsativum* ‘Ctirad’	3×
*H.selago*	19.2	NA	Vejvodová et al. (2024)	OTTO	*Pisumsativum* ‘Ctirad’	4×
*H.selago*	23.2	NA	Vejvodová et al. (2024)	OTTO	*Pisumsativum* ‘Ctirad’	5×
*H.selago*	29.0	NA	Vejvodová et al. (2024)	OTTO	*Pisumsativum* ‘Ctirad’	6×

### ﻿Taxon sampling, DNA extraction, and sequencing

Multiple accessions were sampled for both the new species and its congeneric species. Complete chloroplast genomes were generated by extracting whole genomic DNA from silica gel dried leaf fragments using a modified CTAB approach (Yang et al. 2014). The DNA sample was indexed by tags and pooled together in one lane of a Genome Analyzer (Illumina HiSeq 2000) for sequencing at the Germplasm Bank of Wild Species, Kunming Institute of Botany, CAS (KIB) and Novogene Biotech (Beijing, China).

### ﻿Plastome assemblage and annotation

GetOrganelle toolkit (Jin et al. 2020) and Geneious (https://www.geneious.com) were employed to assemble and annotate newly generated genomes. The previously published plastomes of *Huperzialucidula* (NC_006861) and *H.serrata* (NC_033874) were utilized as reference genomes. The newly sequenced and annotated plastomes were submitted to the GenBank (Table 2). The circular genome plots were generated using Organellar Genome DRAW (Lohse et al. 2013). The boundary of the large-single copy (LSC), small-single copy (SSC), and inverted-repeat (IR) regions for each plastome was visually examined and manually adjusted using Geneious. Plastome characteristics, including genome length and GC content, length of LSC, SSC, and IR region, were compared among all chloroplast genomes from *Huperzia* (Table 2).

**Table 2.** Plastome sequences generated and/or utilized in this study and its characteristics.

Species	Voucher	Locality	GB accession	Genome (GC) LSCSSC IR				Genes Proteins tRNA rRNA			
*H.crassifolia*	LHM2571B	China, Guizhou (Cult.)	OP223752*	153183 (36.3)	103419	19662	30102	131	88	35	8
*H.crassifolia*	ES946-1	China, Hubei, Enshi	OP223753*	153276 (36.3)	103324	19662	30290	131	88	35	8
*H.crassifolia*	YYH15893	China, Hunan, Sangzhi	unpublished^#^	163344 (36.1)	103324	19662	40358	131	88	35	8
*H.javanica*	LHM2743B	China, Yunnan, Lvchun	OP223759*	154275 (36.4)	103982	19669	30624	131	88	35	8
*H.javanica*	LHM2743A	China, Yunnan, Lvchun	OP223760*	154682 (36.4)	104048	19668	30966	131	88	35	8
*H.javanica*	LHM2569B	China, Guizhou (Cult.)	OP223761*	154247 (36.4)	103941	19668	30638	131	88	35	8
*H.lucidula*	Qiu 94173	USA, Wisconsin	MH549639 ^b^	154368 (36.3)	104083	19657	30628	131	87	36	8
*H.lucidula*	Renzaglia 3200	USA, North Carolina	NC_006861 ^c^	154373 (36.3)	104088	19657	30628	119	86	29	8
*H.nanlingensis*	GZY2021012001	China, Guizhou	OP223750*	154146 (36.4)	109852	19668	30626	131	88	35	8
*H.nanlingensis*	LHM2568	China: Guizhou	OP223754*	153806 (36.4)	104038	19668	30100	131	88	35	8
*H.selago*	LHM & HS s.n	Germany, Alps	OP223755*	153518 (36.3)	103901	19667	29950	131	88	35	8
*H.selago*	LHM & HS s.n.	Germany, Alps	OP223756*	153515 (36.3)	103898	19667	29950	131	88	35	8
*H.serrata*	LHM2121	Japan, Mie-ken	OP223757*	153495 (36.3)	104069	19668	29758	131	88	35	8
*H.serrata*	Zhang X.C. 6972	China, Jilin, Helongjiang	NC_033874 ^d^	154176 (36.3)	104080	19658	30438	130	87	35	8
H.serrataf.longipetiolata	R. Wei CBL011	China, Guangdong, Shaoguan	KY609860 ^a^	154415 (36.4)	104120	19667	30628	126	85	33	8
H.cf.sutchueniana	Guo Z.Y. s.n.	China, Guizhou (Cult.)	OP223758*	154697 (36.4)	104053	19678	30966	131	88	35	8
H.cf.sutchueniana	LHM2570	China, Guizhou (Cult.)	OP223751*	154318 (36.4)	104198	19678	30442	131	88	35	8
*P.carinatus*	Anonymous 5309270453	China, Yunnan, Cangyuan	MN566837 ^e^	150349 (34.0)	100582	19455	30312	120	79	33	8
*P.phlegmaria*	Jiang R.H./—	China, unknown	MT786212 ^f^	149711 (33.8)	99862	19465	30384	89	53	30	6

Notes: information given as: species name, voucher information, locality including Nation, Province or Prefecture, and location, and GenBank accession number. Dashes (—) indicate missing data, asterisks (*) indicate the sequences newly obtained in this study. Genomes available already in GenBank were cited with the GenBank accession number and its reference as ^a^ Zhang et al. (2017); ^b^ Mower et al. (2019); ^c^ Wolf et al. (2015); ^d^ Guo et al. (2016); ^e^ Luo et al. (2019); ^f^ Sequence from GenBank. The unpublished whole plastid sequence was kindly shared by Yue-Hong Yan. For the plastome, length of genome as well as the GC content, LSC, SSC and IR region in base pairs (bp) were given. Based on our annotation, the following numbers were reported: number of genes, number of proteins, number of tRNA, and number of rRNA.

### ﻿Phylogenetic reconstruction

Phylogenetic trees were reconstructed using 17 complete chloroplast genome sequences. Among these, 13 plastomes were newly generated that were added by four genomes already available via GenBank. Both Maximum likelihood (ML) and Bayesian inference (BI) were employed in the phylogenetic relationship reconstruction. Models of molecular evolution were determined using jModeltest 2 (Darriba et al. 2012). ML analyses were performed as implemented in RAxML carried our using the web server RAxML-HPC 2 v.8.2.10 (Stamatakis 2014) with 1,000 non-parametric bootstrap replicates (MLBS). Posterior probabilities (PP) were obtained using MrBayes v.3.2.6 (Ronquist et al. 2012) with the model as identified in jModelTest without specifying parameter values.

## ﻿Results

### ﻿Plastome characteristics of *Huperzia*

**Figure 1.**
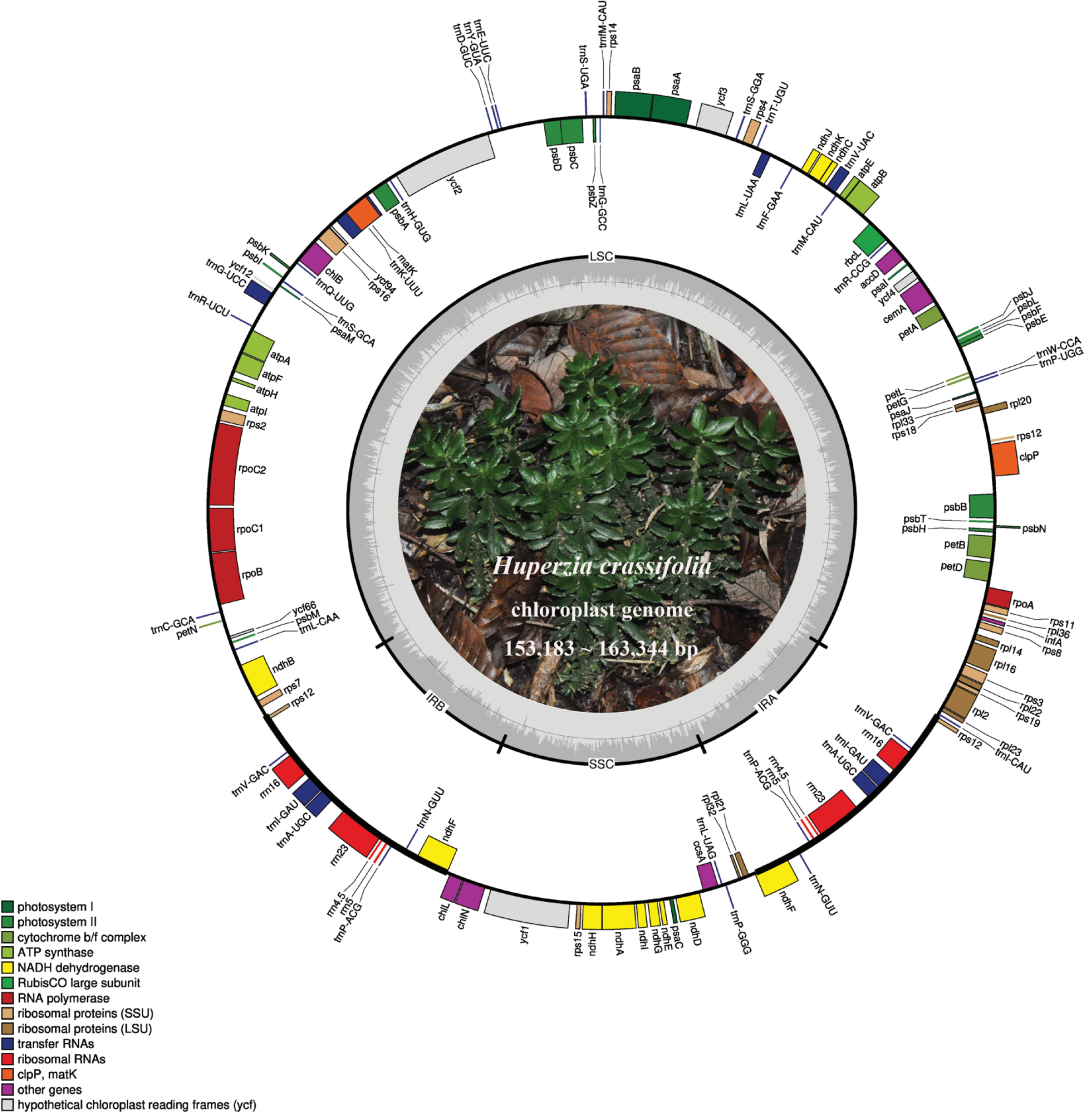
Plastome map of *Huperzia* represented by *H.crassifolia* sp. nov. Genes shown outside the circle were transcribed clockwise, and those inside were transcribed counterclockwise.

The complete chloroplast genome of *Huperzia* possessed the typical quadripartite structure found in most land plants (Fig. 1), including the LSC, SSC, and a pair of IR regions. The assembled genomes contained 131 genes, comprising 88 protein-coding genes, 35 tRNAs, and eight rRNAs. Among the two *H.crassifolia* chloroplast genomes, variation was detected for total length as well as the length of the LSC and IR regions whereas the length of SSC was identical. The 17 *Huperzia* genomes showed some variations in gene order, gene content, and GC content (Table 2).

### ﻿Phylogenetic relationships among *Huperzia* species

**Figure 2.**
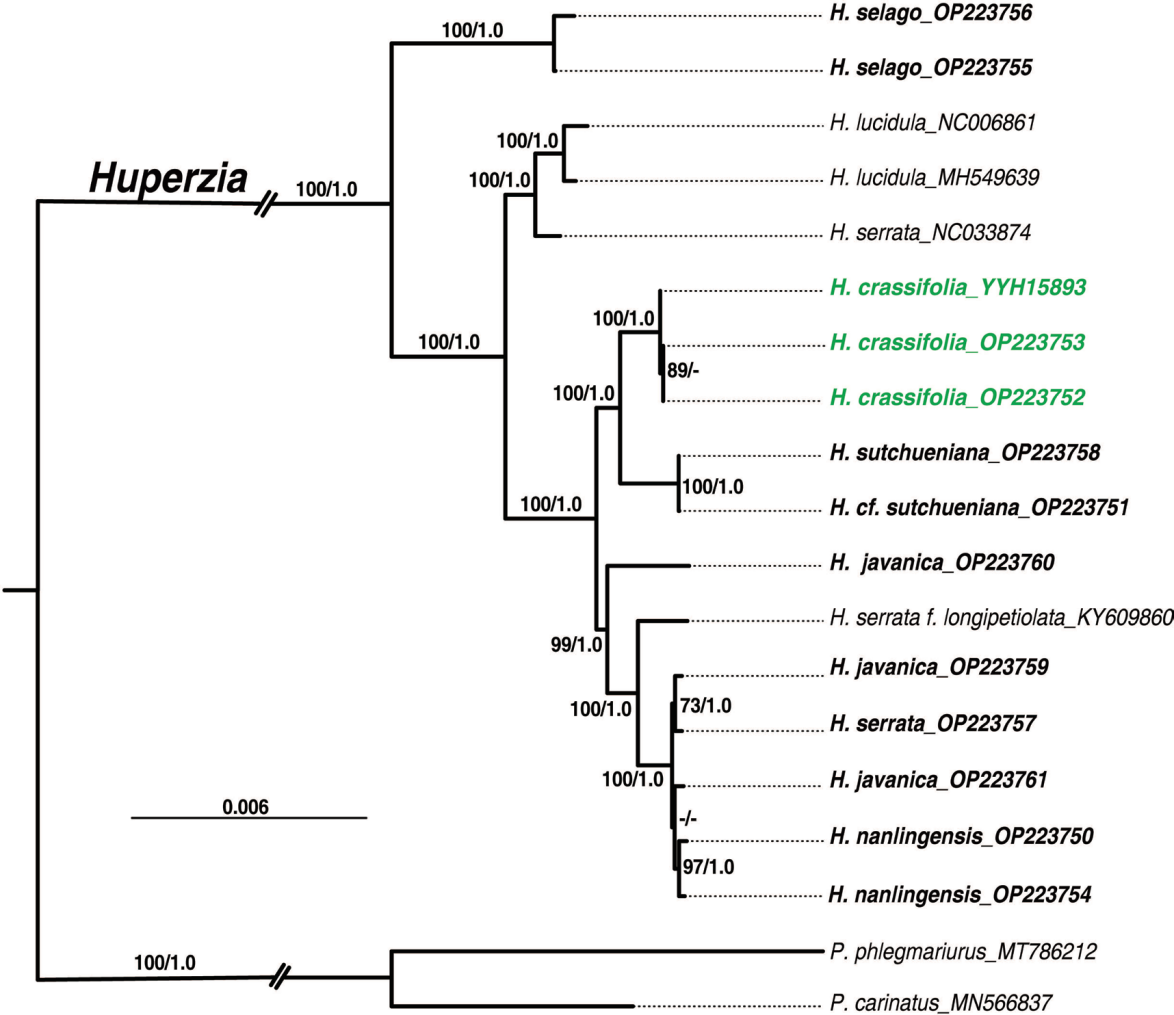
Phylogenetic relationships of *Huperzia* inferred by Bayesian inference (BI) and Maximum likelihood (ML) utilizing 85 CDSs. The consensus phylogram obtained from 1,000 trees sampled in the BI analyses. Branch lengths corresponded to the estimated number of substitution events. Maximum likelihood bootstrap values (MLBS) and posterior probability values (PP) were shown above and/or below the branches, dashes (-) indicate values below 50% (MLBS) and/or below 0.95 (PP). Two species of *Phlegmariurus* were assigned as outgroup taxa.

Alignment of the plastome sequences yielded a matrix of 72,403 positions, in which 4,188 variable sites (5.78%) were identified and 2,768 (3.82%) were parsimoniously informative. Both the complete chloroplast genome sequence and extracted 85 CDSs were used to reconstruct the phylogenetic relationships among the *Huperzia* species. ML and BI analyses of the complete genome sequences and CDSs dataset produced identical tree topologies (Fig. 2). Three accessions of *H.javanica*, the Japanese *H.serrata*, *H.nanlingensis* as well as H.serrataf.longipetiolata were grouped together as a monophyletic clade, whereas the three accessions of *H.crassifolia* were resolved as a well-supported monophyletic entity which formed a sister-taxon relationship to *H.sutchueniana* (Fig. 2).

### ﻿Macro- and micro-morphological comparison

**Figure 3.**
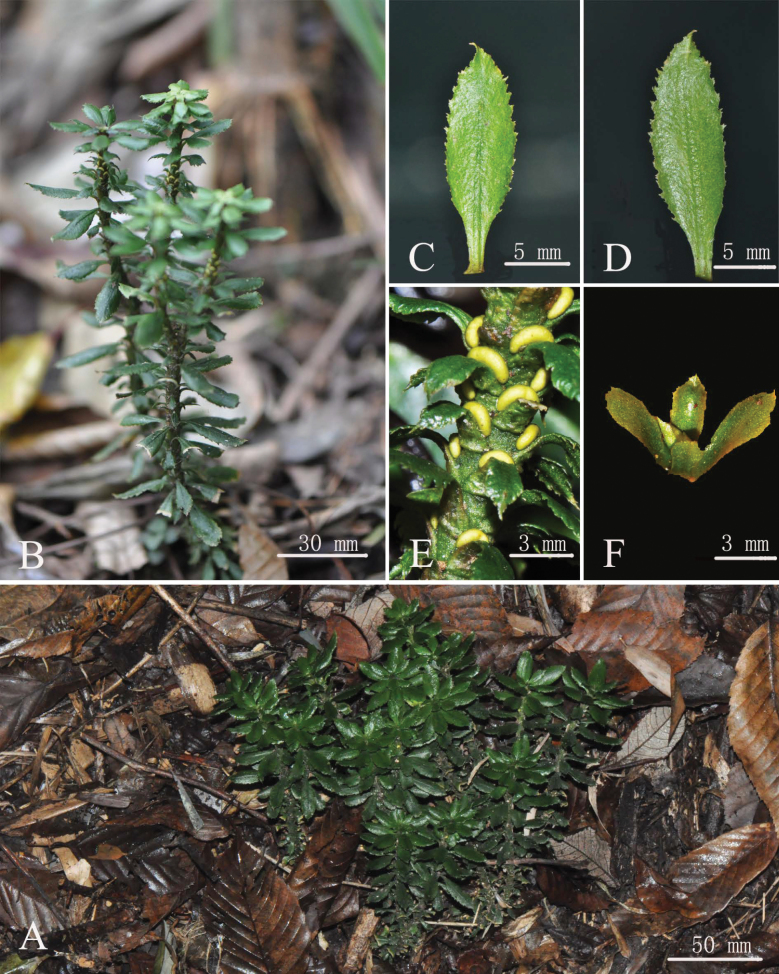
*Huperziacrassifolia* sp. nov. **A, B** habit **C** adaxial view of trophophyll **D** abaxial view of trophophyll **E** fertile portion of the shoot **F** gemmae (Photographed by Zhi-You Guo).

**Figure 4.**
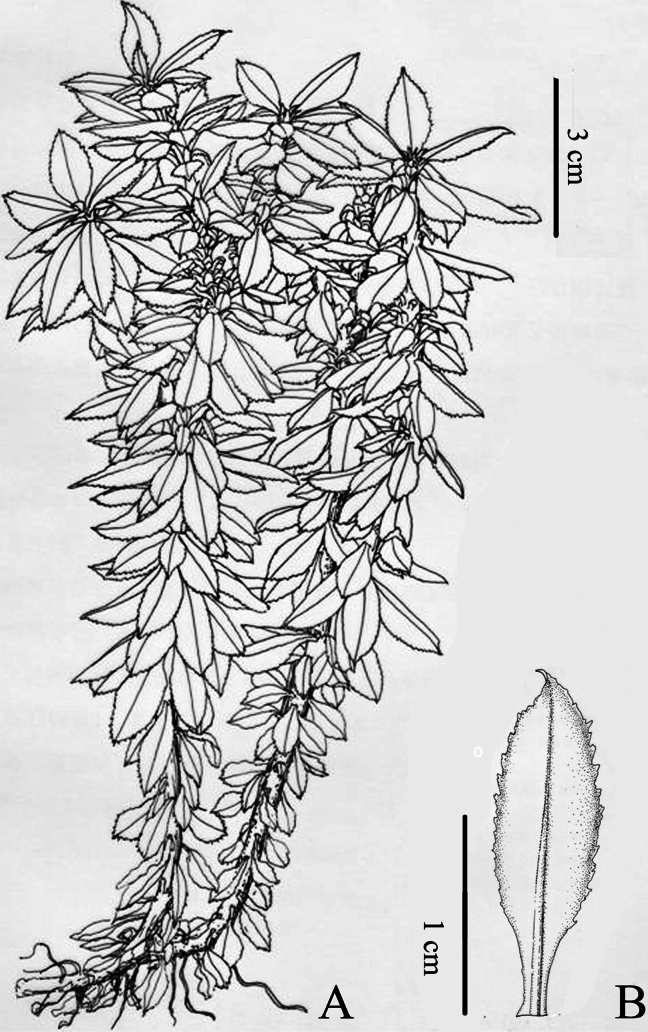
Illustration of *Huperziacrassifolia* sp. nov. **A** habit **B** adaxial view of trophophyll with prominently raised veins and serrate margin. Drawn by Mr. Ying-Bao Sun based on the type specimen Zhi-You Guo 2016022 (QNUN).

**Figure 5.**
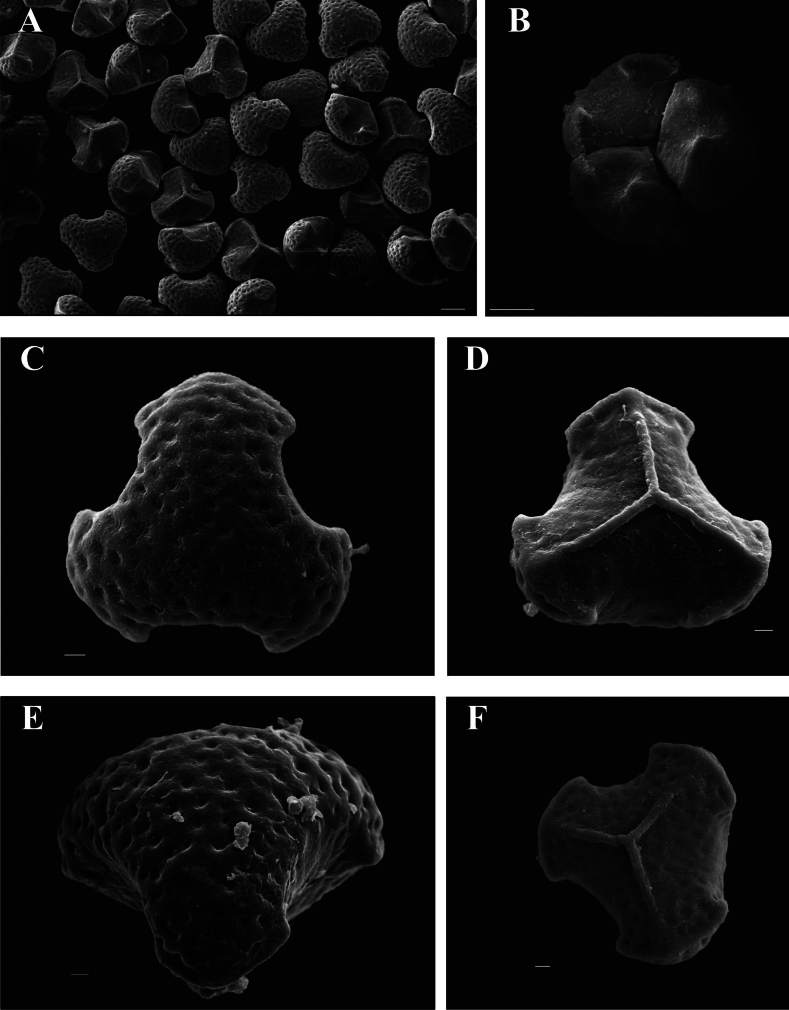
Spores of *Huperziacrassifolia* sp. nov. (**A–D**) and *H.javanica* (**E–F**) **A, B** well-developed trilete spore **C** distal view of the spore **D** proximal view of the spore **E** distal view of the spore **F** proximal view of the spore. Scales bars: 10 μm (**A, B**); 2 μm (**C–F**). Voucher specimen of **A–D**: Liu et al./LHM2571A, voucher specimen of **E, F** Liu ZY/Zhengyu Liu 1708.

The morphological comparison revealed that the new species was similar to the common species *Huperziajavanica* and *H.nanlingensis* in its macro-morphology including leaf shape and serrations on the leaf margin, but other morphological characters distinguished it unambiguously (Figs 3, 4; Table 3). The distinctive characters included round-lanceolate pinnae, thicker coriaceous texture, and slightly involute leaf margins when get dried (Table 3). Besides, plants of *H.crassifolia* have a character of well-differentiated seasonal constriction zones (Fig. 3). *Huperziacrassifolia* has trilete spores with the foveolate ornamentation but differed in the length of laesura besides slight differences in the density of the foveolae when compared with *H.javanica* (Fig. 5).

**Table 3.** Morphological comparison of *Huperziacrassifolia* and its similar species.

Features	*H.crassifolia*	*H.javanica*	*H.nanlingensis*	*H.serrata*	*H.sutchueniana*
Leaf shape	Elliptic-lanceolate	Broadly elliptic-oblanceolate	Elliptic-lanceolate	Lanceolate	Lanceolate
Leaf texture	Thick coriaceous	Thin coriaceous	Coriaceous	Herbaceous	Coriaceous
Leaf margin	Serrate	Serrate	Serrate	Serrate	Sparsely toothed
Serration	Whole part	Whole part	Whole part	Whole part	Upper part
Abaxial vein	Strongly raised	Raised	Raised	Raised	Indistinct
Leaf margin when get dried	Reflexed	Flat	Flat	Flat	Slightly reflexed
Gemmae	Present	Present	Present	Present	Present
Annual constriction zones	Yes	Not prominent	Yes	No	No

Accessions of *Huperziacrassifolia* were distinct in the holoploid genome size from accessions of *H.javanica* and *H.nanlingensis* by having a 2C value of 17.2 =/- SE 0.21 compared to 2C = 8.7 +/- 0.16 and 8.7, respectively (Table 1). Given the holoploid genome size values of the diploid *H.lucidula* and the tetraploid *H.selago*, *H.crassifolia* was interpreted to be a tetraploid.

### ﻿Taxonomic treatment

#### ﻿ Huperzia crassifolia

Taxon classificationPlantaeLycopodialesLycopodiaceae

W.M.Chu & B.Y.Zhang ex Z.Y.Guo sp. nov.

3CE8AFBD-B635-57A8-9E74-AFF9A0148A41

urn:lsid:ipni.org:names:77347495-1

Figs 3 , 4

##### Type.

China • Guizhou Province: Duyun City, Luosike Mountain, between Maozhuchong and Tuanshan, 26.2390, 107.2329, under the broad-leaf forests, alt. 1100–1500 m, 10 March 2016, *Zhi-You GUO 2016022* (Holotype, QNUN!).

##### Diagnosis.

*Huperziacrassifolia* resembled species *H.javanica* in its gross morphology, but can be easily distinguished by the thicker texture and round-lanceolate pinnae. The well differentiated seasonal constriction zones, as well as the dark-green colour of the pinnae, provide further distinctions to species with similar morphology.

##### Description.

Plants terrestrial. Stem erect or ascending, 2–4 times dichotomous branches, 5–25 cm tall, 0.2–0.4 cm in diam. Upper portion of the branches often with numerous gemmae. Leaves sparse, attached at right angles with stem, elliptic to slightly oblanceolate, conspicuously contracted towards base, 1.2–1.9 cm long, 0.4–0.6 cm wide, leathery, shiny, dark green above and light green below, glabrous, midrib conspicuous, base cuneate, decurrent, petiolate, margin straight, slightly reflexed when dry, irregularly serrated, apex acute to slightly cuspidate, teeth acute at apex, coarse; seasonal variation between sporophylls and trophophylls, sporophylls slightly dimorphic by reduced size compared to trophophylls; sporangia attached to the upper side of sporophylls, yellowish, reniform. Spores tetrahedral, with trilete aperture and foveolate ornamentation on distal pole.

##### Etymology.

The specific epithet refers to its thick and coriaceous texture of the pinnae.

##### Vernacular name.

厚叶石杉 (Chinese pinyin: hou ye shi shan).

##### Distribution and habitat.

*Huperziacrassifolia* is a terrestrial firmoss currently only known from Guizhou, Hubei, Hunan, and Chongqing, growing on humus rich soils in shady conditions formed in the undergrowth of broad leaf forests at elevations from 1100 to 1900 m a.s.l.

##### Additional specimens examined.

China • Hubei Province, Shibing County, 5 September 2019, R. K. Li ES946 • Chongqing City, Nanchuan District, Jinfo Mountain, 1500 m, 16 April 1981, *Zhengyu Liu* 1327 (IMC0000082!) • Chongqing City, Nanchuan District, Jinfo Mountain, 1750 m, 15 June 1981, *Zhengyu Liu* 1708 (IMC0000083!) • Chongqing City, Nanchuan District, Jinfo Mountain, 1900 m, 23 October 1978, *Zhengyu Liu* 784137 (IMC0000084!) • Chongqing City, Qianjiang District,1260 m, 23 May 1979, *Zhengyu Liu* 373 (IMC0000081!).

##### Taxonomic notes.

During our studies, the corresponding author had the opportunity to check the specimens collected by taxonomist Zheng-Yu Liu and found that he already noticed the distinctness of *Huperziacrassifolia*, but the taxon had not been properly published. The oldest specimen of *H.crassifolia* carries the species name plus W. M. Chu & B. Y. Zhang as the authorities. Thus, the name has been introduced by these two researchers. To honor their contribution, authorities of this new species are W. M. Chu & B. Y. Zhang *ex* Z. Y. Guo.

##### Conservation status.

The new species *Huperziacrassifolia* is distributed in central and SW China; however the population size of each locality is very small. Especially when one considers the medicinal values of *Huperzia* species, it may reach the stage that it is over-collected. Thus, we tentatively propose *H.crassifolia* as Endangered (EN) according to the IUCN categories and criteria (IUCN 2022).

## ﻿Discussion

Multiple evidence led us to the confirmation that *Huperziacrassifolia* differs from all previously recognized or proposed species. This species is morphologically distinct by its thick coriaceous texture and elliptic to slightly oblanceolate pinnae as well as the well-differentiated seasonal constriction zones. The new species shares with co-occurring members of the *H.javanica* complex the habitat preference to occur in the undergrowth of broad leaf forests.

The morphological variation of this new species has been considered as semi-cryptic with the consequence that this new species was reported as a form of *Huperziajavanica* in some studies (Wang and Pan 2018). The uncertainty caused by limited and somewhat hidden morphological differentiation has been a challenge to the taxonomy of *Huperzia*. One approach to overcome these challenges has been explored in the past by carrying out extensive morphological studies (Shrestha and Zhang 2015b), while this study explored another more powerful approach by integrating morphology and phylogenomics.

Whole chloroplast genomes are supported to be highly effective in providing reliable evidence for species identification in the genus *Huperzia*. Multiple standard chloroplast DNA sequences (our unpublished data as well as previous studies, e.g., Chen et al. 2021) showed that DNA mutation is very low in this old lycophyte lineage and therefore lacks resolution in the species identification. In the current study, we demonstrated that plastome is a powerful approach to investigate species boundaries in the genus *Huperzia*.

Cytological evidence enabling the detection of polyploid taxa has been arguably ignored in the taxonomy of *Huperzia* compared to other land plant groups. However, this is understandable given the reported difficulties in obtaining reliable chromosome counts (Manton 1950) that have been only recently resolved (Vejvodová et al. 2024). Employing genome size measurement may not completely replace the needs of chromosome counts but enables the detection of distinct ploidy levels in lineages of plants that show a highly conserved trend of a positive correlation between chromosome number and genome size such as ferns and lycophytes (Fujiwara et al. 2023; Vejvodová et al. 2024). Applying this approach to the species complex of *Huperzia* is highly promising given the results of the existing chromosome counts suggesting the existence of several ploidy levels (Wagner 1992). Thus, the high cytotype diversity reported for occurrence of the *H.selago* complex in the European Alps (Vejvodová et al. 2024) may be not the exception. This hypothesis is now testable using genome size measurements enabling the screening of many specimens without the need to overcome the technical difficulties of chromosome counting.

Finally, the phylogenetic placement of the new species as sister to *Huperziasutchueniana* raises questions about its origin. The latter species is distinct by the sparsely toothed margin and lanceolate leaves (Table 3) from *H.crassifolia* and *H.javanica*. Together with the genome size indicating *H.crassifolia* as a tetraploid, the conflict between morphology and phylogenetic placement implies the hypothesis that *H.crassifolia* is an allotetraploid originating from hybridisation between *H.javanica* and *H.sutchueniana*. While reticulate evolution has been documented for the firmoss in North America (Wagner 1992), rather less attention has been given to reticulate evolution in the context of the taxonomy of firmoss occurring in China to date.

## Supplementary Material

XML Treatment for Huperzia crassifolia

## ﻿Additional information

### Author ORCIDs

Zhi-You Guo https://orcid.org/0000-0001-5563-3719

Hong-Mei Liu https://orcid.org/0000-0003-2594-9258

Kai-Kai Wang https://orcid.org/0000-0002-0035-2466

Tao Fujiwara https://orcid.org/0000-0002-9397-2831

Xian-Chun Zhang https://orcid.org/0000-0003-3425-1011

Harald Schneider https://orcid.org/0000-0002-4548-7268

### Data availability

The newly generated plastomes have been submitted to the NCBI. All the other data that support findings of this study are available in the main text.
